# The Application of Injectable Hydrogels in Myocardial Infarction Repair: Material Design, Biological Functions and Clinical Translation

**DOI:** 10.3390/ijms27146464

**Published:** 2026-07-21

**Authors:** Zhichao Zhang, Xinyue Lang, Yunlong Zhang, Xufeng Dong, Xiao Han, Chang Xu, Xijing Zhuang

**Affiliations:** 1 Central Hospital of Dalian University of Technology, Dalian 116089, China; zzch@mail.dlut.edu.cn (Z.Z.); langxinyue1010@mail.dlut.edu.cn (X.L.); yunlongzhang@wnmc.edu.cn (Y.Z.); dongxf@dlut.edu.cn (X.D.); hanxiao9935@dlut.edu.cn (X.H.); 2 Faculty of Medicine, Dalian University of Technology, Dalian 116024, China; 3 School of Materials Science and Engineering, Dalian University of Technology, Dalian 116024, China

**Keywords:** injectable hydrogels, myocardial infarction, cardiac tissue engineering, drug delivery

## Abstract

Myocardial infarction remains a major clinical challenge because ischemic injury triggers inflammation, oxidative stress, cardiomyocyte loss, impaired vascularization, and adverse ventricular remodeling. Injectable hydrogels offer a minimally invasive and versatile strategy for post-infarction cardiac repair by providing structural support, localized bioactive delivery, and dynamic modulation of the injured myocardial microenvironment. This review highlights pathology-informed design principles for injectable hydrogels, including shear-thinning injectability, in situ gelation, tissue adhesion, modulus matching, fatigue resistance, biodegradability, and controlled bioactivity. We further discuss hydrogel-based strategies for immunomodulation, angiogenesis, fibrosis attenuation, extracellular matrix remodeling, and myocardial regeneration. Finally, translational progress and key challenges involving biosafety, scalable manufacturing, standardization and long-term efficacy are examined. This review provides a concise framework for developing next-generation injectable hydrogels for myocardial infarction repair and clinical translation.

## 1. Introduction

Myocardial infarction (MI) is one of the leading causes of cardiovascular mortality worldwide and is fundamentally characterized by prolonged myocardial ischemia and hypoxia resulting from acute coronary artery occlusion. Adult cardiomyocytes possess extremely limited regenerative capacity; therefore, ischemia-induced cascade reactions lead to the irreversible apoptosis and necrosis of a large number of cardiomyocytes within a short period. Subsequently, the activated inflammatory response is unable to replenish functional cardiomyocytes, and the necrotic region is ultimately replaced by fibrotic scar tissue [1,2,3]. This pathological alteration further triggers adverse ventricular remodeling, in which the damaged myocardial wall progressively thins under the persistent hemodynamic stress generated by the high-pressure environment of the left ventricle. Consequently, severe local stress concentration develops, exacerbating mechanical injury to cardiomyocytes in the border zone and driving progressive dilation of the left ventricle [4]. This vicious structural cycle markedly reduces myocardial compliance and impairs both systolic and diastolic function, ultimately leading to the irreversible progression toward end-stage heart failure.

Current clinical interventions for MI mainly include pharmacological therapy, percutaneous coronary intervention (PCI), and coronary artery bypass grafting (CABG). Although these approaches can restore myocardial perfusion and delay ventricular remodeling, they are fundamentally defensive strategies that cannot reverse cardiomyocyte loss or repair established scar tissue [5,6]. Cardiac transplantation and ventricular assist devices (VADs), on the other hand, are constrained by practical limitations, including donor shortages and the highly invasive nature of surgical procedures. Therefore, the development of strategies capable of actively modulating the post-infarction myocardial microenvironment, preventing adverse remodeling, and promoting in situ tissue regeneration has become a central challenge in cardiovascular regenerative medicine.

Biomaterial-based tissue engineering strategies have provided new therapeutic opportunities for myocardial repair, among which injectable hydrogels have emerged as a major focus in cardiac tissue engineering owing to their excellent tunability, biocompatibility, and delivery capabilities. Injectable hydrogels are three-dimensional crosslinked polymeric network materials composed of hydrophilic, amphiphilic, or hydrophobic polymers, or combinations thereof, which can be delivered to the target site via syringe or catheter and subsequently form stable gel structures in situ. Compared with conventional implantable materials such as cardiac patches, injectable hydrogels offer significant minimally invasive advantages, enabling precise delivery to the infarcted region and surrounding tissues through catheter-based administration or local injection, thereby reducing surgical trauma and treatment-related risks. Furthermore, their favorable rheological properties and tissue adaptability allow them to conform to the complex morphology of damaged tissues, achieve uniform coverage of the lesion area, and establish stable interfacial integration with the surrounding myocardium, thereby providing essential structural support for subsequent tissue repair and functional restoration.

In addition, the high water content and three-dimensional porous architecture of hydrogels closely resemble those of the native extracellular matrix (ECM). Through advanced molecular design, their mechanical properties and degradation kinetics can be precisely tailored to match the dynamic mechanical environment of the beating heart. Moreover, injectable hydrogels can serve as ideal carriers for the efficient encapsulation and sustained release of stem cells, exosomes, growth factors, and therapeutic agents, thereby achieving dual therapeutic effects through both physical support and biological regulation [7,8]. In recent years, injectable hydrogels have attracted considerable attention in the field of myocardial infarction repair. Their design concept has gradually evolved from a simple biomaterial platform that provides structural support to a multifunctional biomaterial system capable of microenvironmental modulation, promotion of tissue regeneration, and functionalized cargo delivery. Through the rational design of material composition, network architecture, and functional components, injectable hydrogels can not only improve the local microenvironment of injured myocardium but also actively participate in and regulate the tissue repair process.

**Figure 1:**
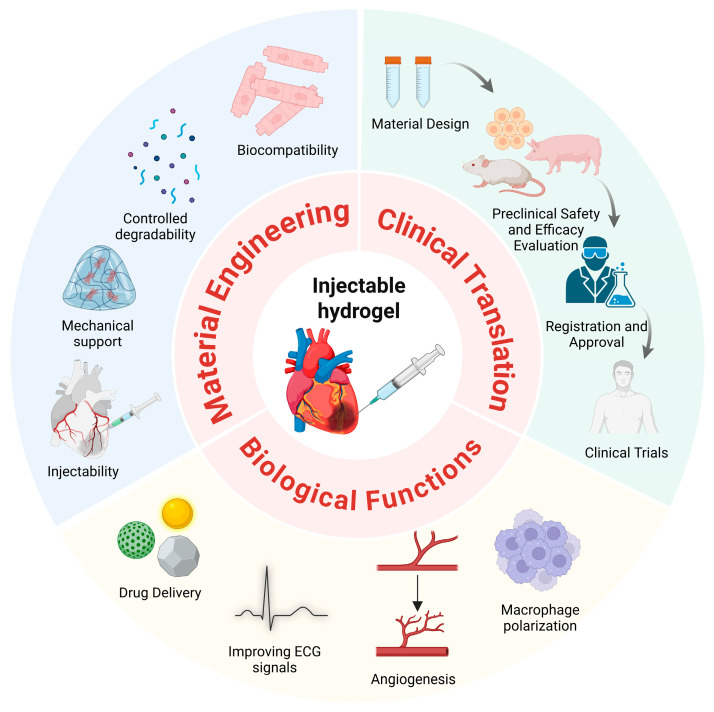
Injectable hydrogel for the treatment of myocardial infarction. Created in BioRender. Zhang, Z. (2026) https://BioRender.com/yyabrwx (accessed on 1 July 2026). Different background colors distinguish the three major aspects of injectable hydrogels: material engineering, biological functions, and clinical translation. The icons represent the corresponding characteristics or evaluation steps indicated by the adjacent labels.

This review systematically summarizes the recent advances in the application of injectable hydrogels for myocardial infarction repair. Focusing on material design and functional engineering, it highlights their therapeutic potential and underlying mechanisms in tissue regeneration (Figure 1). Furthermore, the key challenges associated with their clinical translation are discussed. Finally, future development trends and research directions are outlined, with the aim of providing theoretical guidance for the rational design, optimization, and clinical application of injectable hydrogel-based biomaterials.

## 2. Pathophysiological Mechanisms Following Myocardial Infarction

### 2.1. The Pathological Process of Myocardial Infarction

Myocardial repair following myocardial infarction (MI) is a continuous pathophysiological cascade in which multiple interconnected stages drive the progression from acute injury to heart failure. In the early phase of infarction, local ischemia and hypoxia caused by coronary artery occlusion lead to mitochondrial dysfunction and an energy metabolic crisis. The subsequent intracellular acidosis and pathological burst of reactive oxygen species (ROS) further accelerate cardiomyocyte necrosis and apoptosis [9,10]. The extensive loss of cardiomyocytes results in ventricular wall thinning and stress concentration, which in turn triggers the compensatory activation of neurohormonal systems during the early stage of cardiac injury [11,12].

Subsequently, necrotic cellular debris initiates a sterile inflammatory response characterized by the massive infiltration of neutrophils and pro-inflammatory M1 macrophages, which function to clear damaged tissue remnants [13]. These immune cells also secrete cytokines such as vascular endothelial growth factor (VEGF) to promote early angiogenesis [14]. However, failure to resolve inflammation in a timely manner may result in secondary tissue damage. As the pathological process progresses into the proliferative and reparative phase, the upregulated transforming growth factor-beta 1 (TGF-β1) signaling pathway drives the differentiation of fibroblasts into myofibroblasts [15]. These cells contribute to fibrotic scar formation by finely regulating the expression of matrix metalloproteinases (MMP-2/9) and depositing large amounts of type I and type III collagen. Although the resulting scar tissue temporarily provides mechanical support, its lack of contractile and conductive properties leads to local electromechanical uncoupling [16].

Ultimately, persistent mechanical imbalance and chronic neurohormonal overload maintain myofibroblast activation, promoting the spread of pathological interstitial fibrosis into non-infarcted regions and inducing myocardial hypertrophy [17]. The coexistence of mechanical heterogeneity and electrophysiological conduction disturbances establishes a vicious cycle of adverse ventricular remodeling, which progressively deteriorates cardiac function and ultimately culminates in irreversible end-stage heart failure.

### 2.2. Therapeutic Targets

In response to the stage-specific pathophysiological mechanisms following myocardial infarction (MI), therapeutic strategies are primarily focused on reconstructing the myocardial microenvironment throughout different phases of disease progression. During the early acute injury stage, prompt and proactive reperfusion therapy should be implemented to limit infarct expansion. Simultaneously, excessive local reactive oxygen species (ROS) should be efficiently scavenged, and mitochondrial function should be preserved and restored to mitigate myocardial ischemia/reperfusion injury. During the inflammatory phase, the primary therapeutic objective is to appropriately regulate the inflammatory response, maintaining it within a controlled range and ensuring its timely resolution to prevent secondary tissue damage caused by excessive inflammation. In addition, the delivery of bioactive factors can promote neovascularization and effectively improve the ischemic microenvironment within the infarcted myocardium.

During the proliferative and reparative phase, therapeutic interventions should focus on modulating macrophage polarization, promoting the transition from the pro-inflammatory M1 phenotype to the anti-inflammatory and reparative M2 phenotype, thereby facilitating inflammation resolution and tissue healing. Meanwhile, excessive matrix metalloproteinase (MMP) activity should be suppressed, and collagen deposition should be precisely regulated to prevent excessive extracellular matrix degradation and pathological fibrosis. During the adverse remodeling stage, effective ventricular restraint and mechanical support are required to counteract elevated local wall stress, inhibit early ventricular dilation, and delay long-term remodeling. Furthermore, electrical coupling therapies should be employed to restore synchronized electrical conduction within the infarcted region and reduce arrhythmias associated with chronic conduction heterogeneity. Collectively, these therapeutic strategies aim to alleviate or improve cardiac dysfunction and, for patients who eventually progress to end-stage heart failure, potentially extend the time available before heart transplantation becomes necessary.

## 3. Design Requirements for Injectable Hydrogels

### 3.1. Basic Performance Requirements

#### 3.1.1. Injectability and In Situ Gelation

Injectability and in situ gelation capability are among the most fundamental properties in the design of injectable hydrogels, as they directly influence material delivery, intratissue distribution, and post-gelation retention at the target site [18]. At present, the primary strategies for achieving efficient delivery mainly rely on two mechanisms. The first involves shear-thinning and self-healing networks based on dynamic covalent bonds, such as Schiff base bonds and boronate ester bonds, or non-covalent interactions including hydrogen bonding and electrostatic interactions [19,20]. These networks enable a temporary reduction in viscosity under the high shear stress generated during injection and allow rapid recovery of the gel state once the shear force is removed [21,22]. The second strategy is based on stimulus-responsive sol–gel transition systems, in which crosslinking and gelation are triggered in situ by physiological cues such as body temperature (37 °C), physiological pH, or ionic strength at the target lesion site [23,24].

The trade-off between the injectable time window and in vivo washout remains a major challenge in the design of injectable hydrogels. Excessively rapid gelation kinetics may cause premature solidification of the material within a syringe or minimally invasive catheter, resulting in needle blockage. Conversely, if gelation occurs too slowly, the precursor solution is highly susceptible to displacement and washout by the dynamic blood circulation and interstitial fluid flow present in vivo. Although no universal criterion has been established for in situ gelation time, injectable hydrogels for myocardial repair are generally designed to undergo gelation within seconds to several minutes after administration, depending on the delivery route and gelation mechanism. Consequently, current research efforts are focused on integrating shear-thinning behavior with rapid in situ gelation strategies, enabling injectable hydrogels to maintain excellent flowability during administration while rapidly forming stable gel structures upon reaching the target tissue, thereby minimizing material loss and enhancing local retention efficiency.

#### 3.1.2. Mechanical Matching

The viscoelastic properties of hydrogels are typically characterized by the storage modulus (G′) and loss modulus (G″), and their mechanical performance directly influences structural stability in vivo as well as the ability to support damaged tissues. Traditional design principles emphasize mechanical matching between hydrogels and the native extracellular matrix (ECM) of the target tissue. For the repair of soft tissues such as the myocardium and skin, hydrogels generally require relatively low mechanical moduli to provide adequate compliance and tissue compatibility. In contrast, for load-bearing tissue engineering applications such as bone and cartilage repair, the compressive strength and load-bearing capacity of hydrogels are often enhanced by increasing crosslinking density or incorporating inorganic reinforcing components.

However, a growing body of evidence suggests that static mechanical matching alone is insufficient to meet the dynamic requirements of tissue repair in complex pathological environments. Consequently, mechanically adaptive systems with time-dependent properties have gradually emerged as an important direction in the design of functional hydrogels [25]. In the context of post-infarction ventricular remodeling, for example, the infarcted region undergoes structural weakening due to the rapid degradation of the extracellular matrix and is continuously subjected to elevated ventricular wall stress during the early stage after myocardial infarction [26]. At this stage, hydrogels are required to provide sufficient mechanical support to limit ventricular dilation and maintain local tissue integrity. As the repair process progresses, newly synthesized extracellular matrix is gradually deposited and participates in tissue remodeling. Excessive material stiffness may then restrict the normal contractile activity of the remaining myocardium and interfere with electromechanical coupling. Therefore, an ideal injectable hydrogel should possess dynamic mechanical adaptability, enabling its mechanical properties to be regulated in a controlled manner throughout the tissue repair process. Such a hydrogel could continuously match the evolving requirements of tissue regeneration and facilitate a smooth transition from early structural support to late-stage functional restoration [27].

#### 3.1.3. Biocompatibility and Immunomodulation

Excellent biocompatibility is a fundamental prerequisite for the in vivo application of injectable hydrogels. Both the hydrogel itself and its degradation products should avoid inducing acute or chronic cytotoxicity while also exhibiting favorable hemocompatibility and tissue compatibility, thereby preventing hemolytic reactions, excessive inflammatory responses, and potential genotoxic effects [28,29]. Traditional biomaterial design has primarily emphasized bioinertness, aiming to prolong material retention and maintain functional stability in vivo by minimizing nonspecific protein adsorption and reducing foreign body reactions.

In recent years, with the increasing integration of biomaterials science and immunology, it has become evident that the interaction between implanted materials and the host immune system is not merely antagonistic but rather a dynamic process that can be actively regulated. Consequently, the design philosophy of modern hydrogels has shifted from simply pursuing immune evasion toward the construction of bioactive microenvironments with immunomodulatory functions. Owing to their tunable surface chemistry, hydrophilic–hydrophobic characteristics, and mechanical properties, hydrogels can directly influence the recruitment, adhesion, and phenotypic behavior of immune cells [30,31]. In addition, injectable hydrogels should possess controllable biodegradability to ensure that, after fulfilling their temporary roles in mechanical support or therapeutic delivery, they can be safely degraded and eliminated through non-toxic metabolic pathways [32].

During tissue repair, researchers have incorporated bioactive moieties, immunomodulatory molecules, and functional delivery systems into hydrogels to regulate the polarization state of local macrophages, promoting their transition from a pro-inflammatory phenotype to a pro-regenerative phenotype. This approach suppresses persistent inflammatory responses while enhancing angiogenesis and tissue regeneration [33]. Through the precise modulation of the host immune microenvironment, hydrogels can not only achieve material-level biocompatibility but also actively facilitate tissue repair and functional reconstruction.

### 3.2. Functional Design Requirements

#### 3.2.1. Electrical Conductivity

Following myocardial infarction (MI), electrical isolation between the viable myocardium and the fibrotic scar tissue within the infarcted region is a major cause of malignant ventricular arrhythmias. Therefore, conductive injectable hydrogels have been developed to reconstruct electrical pathways within damaged myocardium. Ideally, the electrical conductivity of conductive hydrogels should be tuned within the 10^−4^ S/cm to closely match the physiological electrical conductivity of native myocardial tissue [34].

To achieve this goal, two main strategies have been widely employed. The first involves incorporating conjugated conductive polymers into the hydrogel network, such as polyaniline, polypyrrole and their oligomers, as well as their graft copolymers. The second strategy is based on embedding conductive inorganic nanomaterials within the hydrogel matrix, including gold nanoparticles, carbon nanotubes, graphene, and MXenes [35,36,37]. These conductive components can establish interconnected electron/ion dual-conduction networks within the inherently insulating hydrogel framework, thereby providing the material basis for subsequent restoration of electrical signal propagation [38].

Despite their excellent electrical conductivity and therapeutic potential, the biosafety of inorganic nanofillers remains an important concern for clinical translation. Gold nanoparticles generally exhibit favorable biocompatibility and chemical stability, although their long-term accumulation in tissues requires further investigation [39]. Carbon nanotubes possess outstanding electrical conductivity but may induce oxidative stress, inflammation, and fibrosis depending on their size, purity, surface functionalization, and dosage [40]. MXene nanosheets have shown promising short-term biocompatibility and antioxidant activity; however, their long-term degradation behavior, biodistribution, and potential toxicity remain largely unknown [41]. In contrast, black phosphorus nanosheets are biodegradable and gradually degrade into phosphate ions, which are generally considered biocompatible. Nevertheless, their rapid degradation and oxidation under physiological conditions may affect material stability and therapeutic efficacy [42]. Therefore, comprehensive evaluations of biodistribution, degradation kinetics, immunogenicity, and long-term toxicity are essential before these conductive nanomaterials can be translated into clinical applications.

#### 3.2.2. Microenvironmental Responsiveness

Following MI, myocardial tissue undergoes a series of dynamic pathological cascade reactions, accompanied by the excessive expression of matrix metalloproteinases (MMPs), bursts of reactive oxygen species (ROS), and local acidosis [43]. These pathological features have inspired the development of diverse stimuli-responsive hydrogel systems based on ROS-, pH-, MMP-, and other endogenous signal-responsive mechanisms [44,45,46,47]. These injectable hydrogels have been developed to recognize and respond to endogenous pathological signals, enabling spatiotemporally controlled degradation, drug release, and regulation of the damaged cardiac microenvironment. By incorporating MMP-sensitive peptide sequences into the polymer backbone or crosslinkers, hydrogels can undergo precisely controlled, on-demand degradation upon exposure to specific pathological enzymes [48]. Meanwhile, the introduction of boronate ester bonds or disulfide bonds can endow hydrogels with excellent ROS responsiveness, enabling bond cleavage in the presence of elevated ROS levels while simultaneously scavenging excessive free radicals in situ, thereby mitigating oxidative stress-induced damage [49,50].

More advanced functional hydrogel designs have gradually evolved from responding to a single stimulus toward the coordinated responsiveness to multiple microenvironmental cues. By integrating pathological signals such as ROS, MMPs, and acidic pH, these hydrogels can achieve precise regulation of degradation behavior, therapeutic cargo release, and tissue repair processes. Such multifunctional responsiveness enhances both the specificity and durability of therapeutic interventions, thereby creating a more favorable local microenvironment for myocardial regeneration.

#### 3.2.3. Controlled Release and Synergistic Delivery of Active Ingredients

Tissue repair following myocardial infarction is a highly dynamic process with distinct temporal characteristics, involving multiple stages, including inflammation resolution, angiogenesis, extracellular matrix remodeling, and tissue maturation [51]. Since these pathological events occur sequentially and require different therapeutic interventions, the rapid and uncontrolled release of a single bioactive agent is often insufficient to achieve effective cardiac repair and may even result in suboptimal therapeutic outcomes. Therefore, injectable hydrogels have been developed as multifunctional delivery platforms capable of precisely controlling the spatiotemporal release of multiple therapeutic agents to address stage-specific pathological changes after MI.

By constructing composite delivery systems that integrate micro- or nanocarriers within macroscopic hydrogel matrices, hierarchical diffusion barriers can be established to precisely regulate the release behavior of bioactive cargos [52]. During the early inflammatory phase, hydrogels can preferentially release anti-inflammatory or antioxidant agents to stabilize the local microenvironment. Subsequently, pro-angiogenic factors such as vascular endothelial growth factor (VEGF) and basic fibroblast growth factor (bFGF) can be continuously released to promote neovascularization and restore tissue perfusion. In the later stages of tissue remodeling, hydrogels can further provide sustained delivery of bioactive components associated with cardioprotection and functional recovery. Through such temporally and spatially controlled release profiles, functional hydrogels can more effectively match the sequential requirements of post-infarction tissue repair and regeneration.

### 3.3. AI-Driven Hydrogel Design

#### 3.3.1. Material Properties and Formulation Prediction

The development of traditional injectable hydrogels has largely relied on empirical trial-and-error approaches, requiring repeated optimization of polymer composition, crosslinking density, and functional components. As a result, the development process is often time-consuming and experimentally costly. In recent years, machine learning (ML) has increasingly been applied to establish predictive models linking hydrogel composition, fabrication parameters, and material performance, thereby enabling rapid property prediction and formulation screening [53]. By integrating parameters such as polymer molecular weight, monomer concentration, crosslinker ratio, shear rate, and drug-loading characteristics, algorithms including random forests (RF), support vector machines (SVM), and artificial neural networks (ANNs) can uncover complex nonlinear relationships among composition, structure, and performance within hydrogel systems.

**Figure 2:**
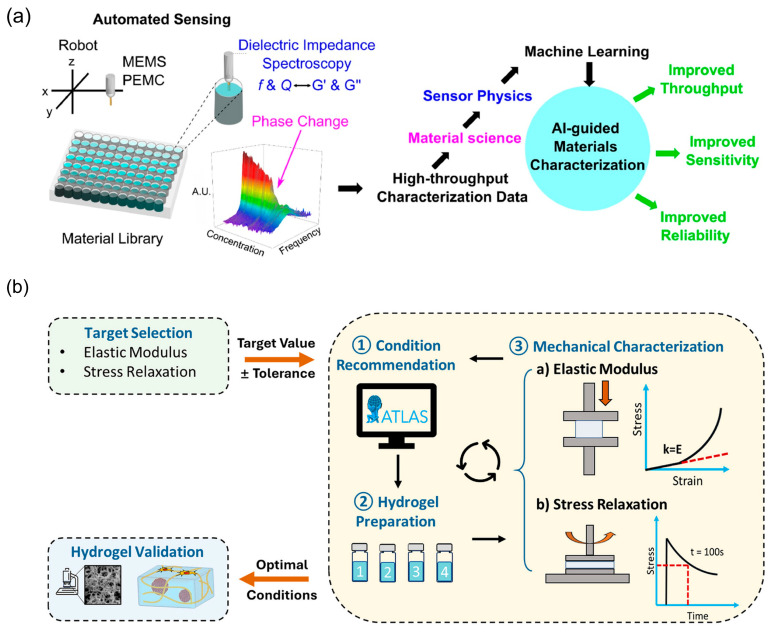
(**a**) Illustration of a rapid, autonomous method for high-throughput characterization (HTC) of hydrogel rheological properties via automated sensing and physics-guided supervised machine learning (spectra shown in arbitrary units (A.U.)). Cropped from Zhang et al., *Appl. Mater. Today* (2023) [54]. (**b**) Workflow of the AL-integrated formulation of tissue-mimetic fibrous hydrogels. Cropped from Chen et al., *Adv. Funct. Mater*. (2026) [55].

As illustrated in Figure 2a, Zhang et al. developed a high-throughput rheological characterization platform based on physics-constrained supervised learning. Through automated data acquisition and machine learning-assisted analysis, the platform enabled rapid prediction of hydrogel viscoelastic parameters, achieving an approximately 70-fold increase in testing efficiency compared with conventional rheological measurements [54]. Such data-driven design strategies facilitate the establishment of composition–structure–property relationships in hydrogels and provide valuable guidance for the development of injectable hydrogels that simultaneously exhibit favorable injectability, appropriate gelation kinetics, and mechanically compatible properties for tissue repair.

#### 3.3.2. Active Learning and Intelligent Customization

For emerging functional hydrogel systems, the limited availability of experimental data and the vast formulation space often restrict the performance of conventional machine learning models. In recent years, closed-loop design strategies that integrate active learning (AL) with Bayesian optimization (BO) have gradually been introduced into hydrogel development. Through an iterative workflow of “model prediction–experimental validation–data feedback,” these approaches enable the rapid optimization of material properties.

Chen et al. incorporated active learning and multi-objective Bayesian optimization into the design of fibrous hydrogels. Using only a limited experimental dataset, they achieved precise regulation of key viscoelastic parameters, including elastic modulus and stress relaxation behavior, and successfully identified fibrous hydrogel formulations capable of mimicking the mechanical microenvironments of extracellular matrices (ECMs) in different tissues [55] (Figure 2b). This study established a digital mapping relationship among material composition, fabrication parameters, and macroscopic mechanical properties while substantially reducing the number of experimental screening cycles required. In the context of myocardial repair, such active learning-based inverse design strategies hold great promise for the rapid identification of injectable hydrogel formulations whose mechanical properties closely match those of native myocardial tissue, thereby improving material development efficiency and reducing reliance on empirical trial-and-error approaches.

## 4. Functional Injectable Hydrogels for MI Repair

Before discussing representative hydrogel systems, it should be noted that most of the studies summarized in this section were conducted in rodent myocardial infarction models. While these models provide valuable insights into hydrogel design and therapeutic mechanisms, substantial differences in cardiac anatomy and physiology between rodents and humans may influence hydrogel performance and therapeutic outcomes. Therefore, further validation in large-animal models and clinical studies remains essential for successful clinical translation.

### 4.1. Material Platforms

**Figure 3:**
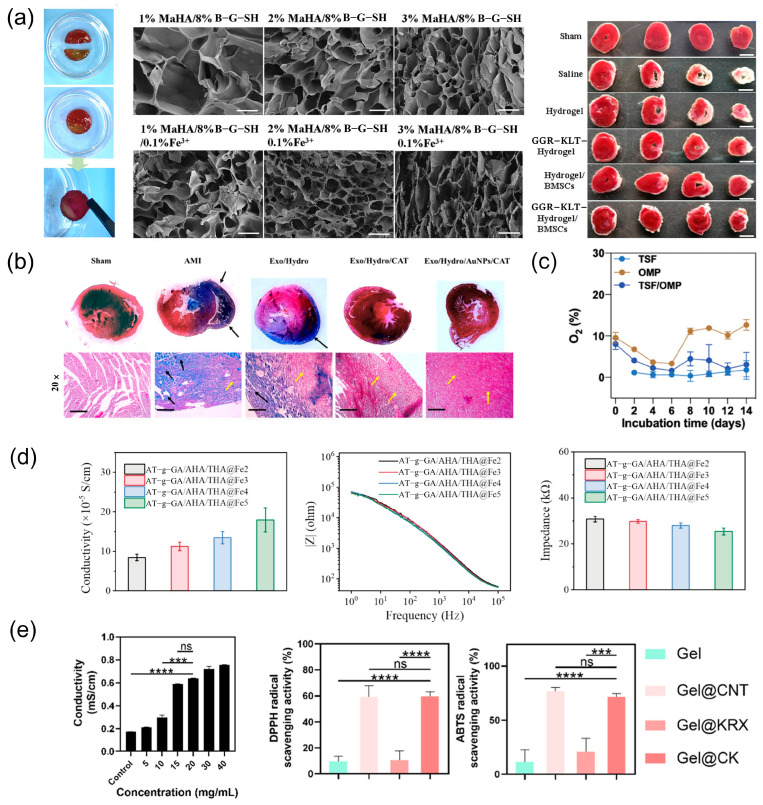
(**a**) Self-healing of MaHA/B-G-SH/Fe3+ hydrogels, SEM and TTC staining of SD rat hearts. Scale bars: 100 μm for all SEM images and 2 mm for all TTC-stained heart sections. Adapted from Zheng et al., *J. Control Release* (2022) [56]. (**b**) Representative photomicrographs of Masson-stained myocardium of control, AMI, Exo/Hydro, Exo/Hydro/CAT, and Exo/Hydro/AuNPs/CAT with different magnifications. Scale bar = 50 µm for 20×. Cropped from Xu et al., *J. Nanobiotechnol.* (2025) [57]. (**c**) Oxygen release kinetics of hydrogels containing different concentrations of SDF or OMP. Cropped from Hassan et al., *Small* (2024) [58]. (**d**) The electrochemical activity of hydrogels includes electrical conductivity, impedance spectroscopy, and interfacial impedance at 20 Hz. Adapted from Yu et al., *J. Control Release* (2026) [59]. (**e**) Conductivity and Radical Scavenging Efficiency of Hydrogels (**** *p* < 0.001, *** *p* < 0.005, with “ns” denoting no significant difference). Cropped from Luo et al., *ACS Appl. Mater. Interfaces* (2025) [60].

Natural polymeric materials, including alginate, chitosan, hyaluronic acid, collagen, gelatin, silk fibroin, and decellularized cardiac extracellular matrix, possess excellent biocompatibility, extremely low host immunogenicity, and abundant endogenous cell-adhesion sites owing to their chemical similarity to native extracellular matrices in vivo. For example, Zheng et al. [56] developed an integrated dual-crosslinked dynamic conductive hydrogel (MaHA/B-G-SH/Fe^3+^) based on modified hyaluronic acid and gelatin, in which ionic interactions and chemical covalent bonds were combined to construct the hydrogel network. As shown in Figure 3a, the hydrogel exhibits excellent self-healing behavior and promotes effective myocardial repair, as evidenced by histological staining of infarcted rat heart tissue. 

In addition, Blended systems composed of chemically modified alginate and fibrin macromolecules, achieved through oxidation or the introduction of specific ionic coordination bonds [57], can form dense interpenetrating polymer networks (IPNs) between linear polymer chains, thereby effectively attenuating the progression of acute inflammation during the early stage of myocardial infarction (Figure 3b).

Despite their excellent biocompatibility, natural polymer-based hydrogels often suffer from limited mechanical strength and tunability, which has motivated the development of synthetic polymer-based systems. One of the greatest advantages of synthetic polymers lies in the tunability of their molecular backbones, which allows specific bioactive moieties or stimuli-responsive covalent bonds to be chemically integrated directly into the polymer chain. Following this design principle, Yang et al. [61] copolymerized thermoresponsive polypropylene glycol (PPG) blocks, redox-active diselenide-containing units (DH-Se), and hydrophilic polyethylene glycol (PEG) segments to construct a novel injectable selenium-containing polyurethane hydrogel network, poly(DH-Se/PEG/PPG urethane). By exploiting the intrinsic anti-inflammatory and anti-fibrotic properties of selenium, this material achieved autonomous improvement of cardiac function in a murine myocardial infarction model without the incorporation of any exogenous therapeutic agents. To further enhance mechanical stability under dynamic shear stress, multiple studies have developed multicomponent synthetic copolymer networks [62,63]. By introducing highly elastic supramolecular crosslinks, hydrogen-bonding clusters, or hydrophobic association domains, these hydrogels exhibit superior fatigue resistance and dynamic self-healing capability, enabling them to maintain structural integrity and sustained mechanical support throughout tens of thousands of high-frequency cardiac contraction–relaxation cycles.

While natural and synthetic hydrogels provide the fundamental material basis, their inherent limitations in functionality have driven the development of composite hydrogel systems with enhanced structural and therapeutic performance. Hassan et al. [58] innovatively reported a silk-based hybrid hydrogel system (Figure 3c). By incorporating oxygen-releasing micro/nanoparticles and matrix-bound angiogenic factors (SDF-1α) into the hydrogel, the system achieved long-term, stable, and synergistic delivery under the dynamic mechanical environment of cardiac contraction. Other approaches have embedded nanocapsules, liposomes, or mesoporous particles into hydrogel networks to form diffusion barriers, thereby improving the stability and in vivo retention of bioactive factors [64,65]. In addition, two-dimensional nanosheets such as black phosphorus and MXenes have been used as reinforcing units to enhance hydrogel mechanical properties while introducing additional functions including conductivity, photothermal activity, and ROS scavenging [66]. Similarly, MOF-based systems (e.g., ZIF-8) have been incorporated as drug reservoirs [67], enabling sustained release of therapeutic agents and bioactive metal ions to promote angiogenesis and endothelialization.

Overall, different hydrogel platforms exhibit distinct advantages and limitations. A comparative summary of the major advantages and limitations of different hydrogel platforms for myocardial infarction repair is provided in Table 1.

### 4.2. Mechanical Support and Ventricular Remodeling

**Figure 4:**
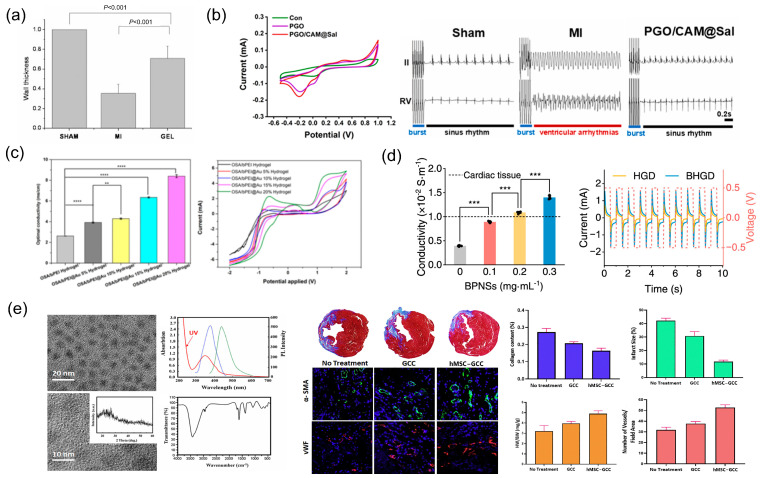
(**a**) Histological analysis of the infarct size and wall thickness. Cropped from Yoon et al., *J. Biomed. Mater. Res. B Appl. Biomater.* (2009) [68]. (**b**) CV curves of different hydrogels and burst pacing-induced supraventricular tachycardia (VA) images in PGO/CAM@Sal hydrogel-treated hearts. Cropped from Song et al., *Mater. Today Bio* (2026) [70]. (**c**) Electrochemical behavior of hydrogels in different groups (** *p* < 0.01, **** *p* < 0.0001). Cropped from Wang et al., *Chem. Eng. J.* (2026) [71]. (**d**) Electrical properties of the hydrogels, including conductivity, current injection curves under biphasic stimulation, and charge injection capacity (*** *p* < 0.001). Cropped from Qiu et al., *ACS Appl. Mater. Interfaces* (2024) [72]. (**e**) Preparation of GQDs and their role in the heart. Scale bars: 20 nm in the upper-left TEM image and 10 nm in the lower-left TEM image. Cropped from Si et al., *J. Photochem. Photobiol. B* (2020) [73].

Following myocardial infarction, extensive cardiomyocyte death leads to the gradual replacement of the injured region by non-contractile fibrotic scar tissue, which in turn results in infarct wall thinning and progressive ventricular dilation. To prevent this adverse ventricular remodeling, early studies primarily focused on exploiting the physical space-filling effect of injectable hydrogels. For example, as shown in Figure 4a, direct injection of a basic hyaluronic acid hydrogel into the infarcted region was reported to significantly increase infarct wall thickness by up to 200%, thereby effectively reducing wall stress and promoting functional recovery [68]. Similarly, an alginate/hyaluronic acid composite hydrogel encapsulating lyophilized platelet-rich fibrin was shown to enhance ventricular wall strength while simultaneously replenishing the local microvascular network within the extracellular matrix [69].

Building on this concept, decellularized porcine myocardial extracellular matrix hydrogels chemically crosslinked via thermosensitive reactions or genipin–chitosan coupling have been widely developed to better recapitulate the complex native myocardial extracellular matrix. These materials not only exhibit elastic moduli comparable to native myocardium but also demonstrate excellent long-term dynamic remodeling capability, persisting for more than 12 weeks [74]. In addition, combination therapy using methylglyoxal scavengers such as fisetin together with type I collagen hydrogels has been shown to effectively inhibit adverse ventricular remodeling by reducing advanced glycation end-product accumulation, significantly decreasing scar size and improving cardiac pumping function [75].

### 4.3. Conductive Hydrogels for Electromechanical Integration

#### 4.3.1. Design Strategies

The first strategy for imparting electrical conductivity to hydrogels involves grafting conjugated conductive polymers, such as polyaniline (PANI), polypyrrole (PPy), or their oligomers, onto traditional flexible polymer backbones. As shown in Figure 3d, Yu et al. [59] constructed a supramolecular hydrogel by combining gelatin grafted with an aniline tetramer and adipic dihydrazide-modified hyaluronic acid, while introducing a coordination complex formed between 2,3,4-trihydroxybenzaldehyde and iron ions. The system was crosslinked via dynamic Schiff base bonds and simultaneously encapsulated L-arginine, thereby bridging the non-conductive infarcted region in vivo, stabilizing ventricular electrophysiology, and significantly reducing susceptibility to arrhythmias.

By integrating polyaniline oligomers into multidimensional network structures [76], and precisely tuning the grafting density of conductive segments, the electrical conductivity of hydrogels can be adjusted within the 10^−4^ S/cm, which closely matches the physiological conductivity of native myocardial tissue [77].

The second strategy does not rely on complex organic synthetic modifications; instead, it directly incorporates inorganic nanomaterials with intrinsically high electrical conductivity into the hydrogel matrix. Xu et al. [57] introduced gold nanoparticles (AuNPs) into an alginate–fibrin network to construct a biohybrid conductive system. In addition, studies employing one-dimensional carbon nanotubes (CNTs) or two-dimensional graphene derivatives as conductive dopants [60,78] have demonstrated that these high-aspect-ratio or high-surface-area nanounits not only form dense electron transport networks but also enabling reactive oxygen species (ROS) scavenging functionality (Figure 3e).

For example, electrostatic complexation between negatively charged inorganic nanounits and positively charged polymer backbones can achieve efficient electrical integration even at very low inorganic loading levels. The advantage of inorganic nanomaterials lies in their high conductivity and excellent physicochemical stability. Through targeted or surface charge modification, these materials can simultaneously provide conductive bridging functions and, owing to their rigid nanoscale architecture, enhance the shear resistance and mechanical support of hydrogels for the infarcted ventricular wall.

#### 4.3.2. Therapeutic Outcomes

Researchers have successfully integrated polypyrrole (PPy) [70,79], gold nanoparticles (AuNPs) [71], black phosphorus nanosheets (BPNSs) [66,72], and photoluminescent carbon dots [73] into hydrogel networks, as shown in Figure 4b–e. These conductive hydrogels effectively bridge electrically isolated infarcted regions, restore synchronized electrical signal propagation between cardiomyocytes, significantly reduce post-infarction ventricular arrhythmias, and improve left ventricular synchronous contraction function. Meanwhile, considering the continuous high-frequency contraction–relaxation motion of the heart, conductive hydrogels are required to exhibit excellent dynamic mechanical stability. Smart networks constructed via dynamic covalent bonds formed through Schiff base reactions between chitosan-grafted dihydrocaffeic acid and oxidized pullulan [80], or via Michael addition reactions between multi-arm conductive crosslinkers such as tetraphenylamine–poly(ethylene glycol) diacrylate (TA-PEG) and sulfated hyaluronic acid (HA-SH) [81], endow the materials with excellent shear-thinning behavior, self-healing capability, and robust fatigue-resistant electrical conductivity, enabling structural integrity maintenance without leakage under continuous large-deformation cyclic loading.

In summary, the design of modern conductive hydrogels has evolved from simple electrical signal bridging to an integrated multifunctional platform combining dynamic mechanical adaptability and self-healing capability. Despite their promising ability to restore electrical coupling, the long-term biosafety, degradation behavior, and potential accumulation of conductive nanomaterials remain important considerations for clinical translation. Furthermore, ensuring stable electrical performance under dynamic cardiac conditions, minimizing potential immune responses, and establishing scalable and reproducible manufacturing processes are critical prerequisites for advancing conductive hydrogels toward clinical applications. Future research should therefore focus on optimizing the integration of electrical conductivity, mechanical compatibility, and biological safety to develop clinically applicable conductive hydrogel platforms for myocardial repair.

### 4.4. Drug and Biomolecule Delivery Hydrogels

**Figure 5:**
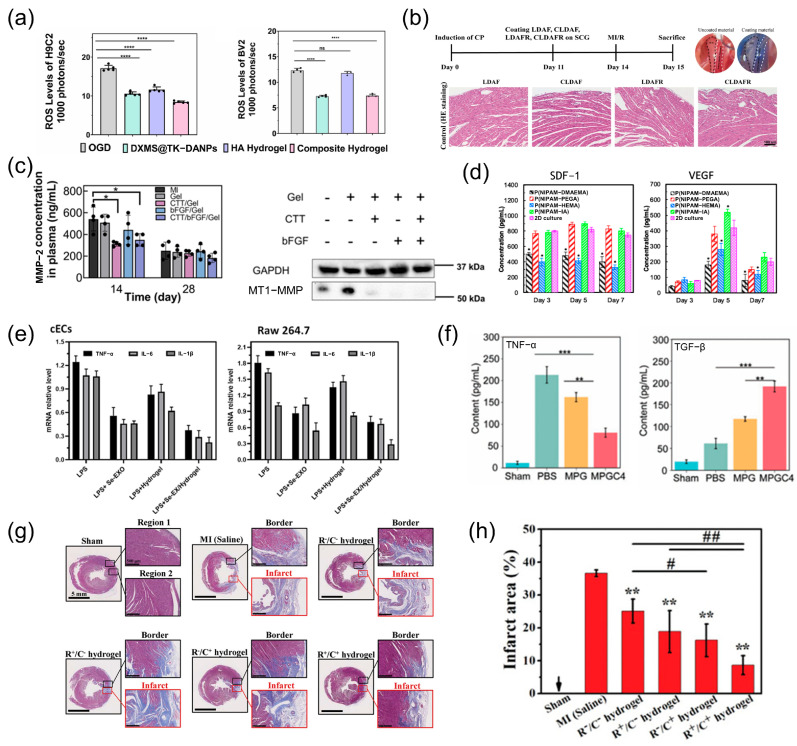
(**a**) In vitro ROS expression in H9C2 cells and BV2 cells when exposed to oxygen-glucose deprivation (OGD) (**** *p* < 0.001, with “ns” denoting no significant difference). Cropped from Wang et al., *Compos. Part B Eng.* (2024) [82]. (**b**) Representative images of hematoxylin–eosin staining: The material was not toxic to the myocardium. Scale bar = 100 µm. Cropped from Wu et al., *J. Control Release* (2025) [85]. (**c**) Dynamic change in MMP2 in plasma and immunoblotting of α-actinin and MT1-MMP from in vivo tissues (* *p* < 0.1). Cropped from Niu et al., *Acta Biomater.* (2025) [52]. (**d**) Growth factors released from hCSCs in the P(NIPAM)-based microgel network and 2D culture at days 3, 5, and 7 (* *p* < 0.05). Adapted from Cui et al., *ACS Appl. Mater. Interfaces* (2018) [86]. (**e**) mRNA expression level of pro-inflammatory cytokines (TNF-α, IL-6 and IL-1β) on LPS-induced cECs and Raw 264.7. Adapted from Lin et al., *J. Drug Deliv. Sci. Technol.* (2023) [87]. (**f**) Quantitative analysis of the secretion of M1 (TNF-α) and M2 (TGF-β) macrophage-related factors by ELISA 3 days after MI (** *p* < 0.01, *** *p* < 0.001). Adapted from Chen et al., *Adv. Mater.* (2023) [88]. (**g**) Masson’s trichrome staining of the whole heart and representative images in the infarct and border zones. Scale bar = 5 mm. Cropped from Ding et al., *Small* (2020) [89]. (**h**) Quantitative analysis of infarcted area in the whole heart (*n* = 5). Normal hearts from the Sham group were also stained as a comparison (** *p* < 0.01 versus MI (Saline) group; # *p* < 0.05 and ## *p* < 0.01). Cropped from Ding et al., *Small* (2020) [89].

Due to severe damage to the local microvascular network in the infarcted myocardium, systemic administration often fails to effectively reach the core region of the lesion. Injectable hydrogels, owing to their highly interconnected porous structures and tunable matrix interactions, have emerged as ideal carriers for localized drug delivery. By pre-encapsulating therapeutic agents within polymeric nanoparticles, liposomes, or micelles prior to incorporation into hydrogels, multi-stage sustained release can be achieved while protecting labile drugs from degradation. Representative systems include ROS-responsive TK-DA nanoparticles loaded with dexamethasone combined with an ECM-mimicking dynamic hydrogel crosslinked via boronate ester bonds [82] (Figure 5a), chitosan thermosensitive hydrogels encapsulating antioxidant α-tocopherol (AT) liposomes to suppress oxidative stress-induced cardiomyocyte injury [83], and collagen/fucoidan hydrogels incorporating polyvalent polyphenol nanoparticles (ESaB NPs) combining enalaprilat and salvianolic acid B. These designs can effectively inhibit the pathological activation of the local renin–angiotensin–aldosterone system (RAAS) and promote cardioprotection [84].

In addition, in the context of chronic pain-associated myocardial ischemia–reperfusion injury, a composite hydrogel system (CLDAFR) encapsulating celecoxib and ropivacaine can be precisely injected into the superior cervical ganglion (SCG), enabling sustained drug release to reduce neuronal inflammation and sympathetic overactivation, thereby providing a novel systemic regulatory approach for the treatment of ischemia–reperfusion injury (Figure 5b) [85]. From a microstructural biomimetic perspective, co-loading stromal cell-derived factor-1 (SDF-1) nanoparticles and vascular endothelial growth factor (VEGF) nanoparticles into chitosan/gelatin hydrogels can effectively mimic the nanoscale organization of native extracellular matrix, synergistically promoting endothelial and smooth muscle cell migration and accelerating neovascularization [90].

For vascular endothelial growth factor (VEGF) and other proteins that are prone to rapid diffusion and inactivation, simple physical blending often leads to rapid drug loss. Covalent conjugation of VEGF using aliphatic polyester thermosensitive hydrogels (HG) can enable long-lasting, localized, and non-denaturing VEGF release confined to the infarct border zone, thereby promoting ischemic cardiac regeneration [91]. In addition, the design of a bifunctional EBP-PR1P targeting peptide enables specific tethering of VEGF to injectable cardiac ECM hydrogels, which preserves its bioactivity while sustaining activation of downstream VEGF–Akt signaling pathways [92]. For multi-factor synergistic systems, local delivery of basic fibroblast growth factor (bFGF) can simultaneously attenuate cardiac fibrosis and promote myocardial vascularization [93]. Furthermore, co-delivery of an MMP-2-specific inhibitory peptide (CTT) with bFGF can synergistically block extracellular matrix degradation via specific peptide network modifications while effectively enhancing endothelial cell migration and angiogenesis (Figure 5c) [52].

This stepwise evolution from single-factor release to multi-drug co-delivery enables precise therapeutic coverage across different pathological stages of myocardial infarction. For example, to address the severely limited regenerative capacity in chronic myocardial infarction (CMI), a biomimetic hydrogel system co-delivering the stem cell homing factor SDF-1 and the angiogenic peptide Ac-SDKP can synergistically recruit endogenous stem cells, enhance mature vascular formation, and significantly reverse the progression of chronic heart failure within four weeks [94]. In addition, a hybrid system based on oxidized sodium alginate crosslinking gelatin nanoparticles encapsulating BIO and IGF-1 enables sustained co-delivery of these potent factors, stimulating endogenous cardiomyocytes to undergo dedifferentiation and proliferative regeneration in situ [95].

Drug-loaded hydrogels have relatively high translational potential because they can utilize clinically approved therapeutic agents; however, achieving precise spatiotemporal release and maintaining drug stability during manufacturing remain major challenges.

### 4.5. Cell and Exosome Delivery Hydrogels

Exogenous cell transplantation was once considered the most direct strategy for reconstructing damaged myocardium and promoting regeneration. However, conventional liquid-phase injection is associated with extremely low cell retention and severe trigger-induced cell death caused by ischemia and oxidative stress. Injectable hydrogels, as three-dimensional artificial extracellular matrices, can provide a relatively safe hydrophilic buffering microenvironment for transplanted cells. By finely tuning the surface charge and hydrophilicity of NIPAM-based microgels, the therapeutic function of cardiac stromal cells (CSCs) can be effectively regulated, maintaining high cell viability and promoting the in situ formation of three-dimensional cell spheroids. This substantially improves cell retention and stimulates robust secretion of factors such as VEGF and IGF-1 [86], as shown in Figure 5d. Meanwhile, a bioactive chitosan/collagen hydrogel matrix incorporating conductive carbon dots can further encapsulate and enhance the survival of human bone marrow mesenchymal stem cells (hMSCs) in situ, as well as upregulate the expression of pro-angiogenic markers [73]. In addition, co-application of mesenchymal stem cells with a collagen/hyaluronic acid hydrogel loaded with nano selenium–ruthenium (Se–Ru NPs) enables strong antioxidant protection. This reduces LPS-induced apoptosis of H9c2 cardiomyocytes by approximately fourfold and significantly enhances the retention of viable cells in ischemic cardiac tissue, as well as increasing ventricular wall thickness [96].

Although hydrogels partially improve cell survival, the inherent limitations of living cell transplantation—including immune rejection, tumorigenic risk, and stringent quality control requirements—remain major barriers to clinical translation. In this context, the field is undergoing a paradigm shift toward a cell-free paracrine therapy approach. Shear-thinning hydrogels can serve as efficient delivery platforms for sustained release of extracellular vesicles (EVs)/exosomes derived from endothelial progenitor cells (EPCs), significantly enhancing angiogenesis and improving post-infarction cardiac function recovery [97]. Similarly, injectable conductive and biodegradable hydrogels enhanced with black phosphorus for delivery of adipose-derived stem cell (ADSC)-derived exosomes have demonstrated excellent myocardial repair effects while effectively preventing arrhythmias [66]. Furthermore, a collagen/tannic acid composite hydrogel loaded with mesenchymal stem cell-derived exosomes (EX) and selenium nanoparticles (Se NPs) exhibits strong anti-inflammatory activity and promotes endothelial cell migration (up to 95%) while downregulating pro-inflammatory factor expression by approximately threefold within three weeks [87] (Figure 5e). In addition, alginate hydrogels loaded with Annexin A1 [98] or phosphatidylserine-modified liposome-based “artificial apoptotic cell/VEGF” composite hydrogels [99] can mimic the immunogenic features of naturally apoptotic cells. These systems modulate macrophage phenotype via the AMPK–mTOR signaling axis, promoting polarization toward the pro-reparative M2 phenotype and thereby effectively attenuating post-infarction cardiac remodeling.

Although cell-laden hydrogels have demonstrated encouraging regenerative outcomes, their clinical application is still limited by poor cell survival, high manufacturing costs, and complex regulatory requirements associated with cell-based therapies.

### 4.6. Gene Delivery Hydrogels

Gene therapy can regulate local cellular gene expression at the molecular level, offering unique advantages in promoting angiogenesis and inhibiting adverse ventricular remodeling. However, free nucleic acid molecules are highly susceptible to enzymatic degradation in physiological environments, and their non-targeted distribution may lead to systemic toxicity. Injectable hydrogels can effectively confine nucleic acids within the infarct border zone, with the core design relying on functionalized nanocarriers for in situ immobilization and protection of genetic cargo.

For example, microRNA-29b mimics with anti-fibrotic effects can be encapsulated in mesoporous silica nanoparticles coated with tannic acid/zinc ion (TA/Zn) complexes and further embedded into alginate hydrogels. This system enables a cascade process of initial ROS scavenging followed by nucleic acid release, thereby effectively inhibiting cardiac fibroblast activation and collagen deposition [100]. To promote endogenous regeneration, shear-thinning hydrogels loaded with microRNA-encapsulated polymeric nanoparticles (miNPs) have been shown to successfully trigger cardiomyocyte and endothelial cell proliferation, increasing ejection fraction from 45% to 64% at 4 weeks, reducing scar area from 20% to 10%, and doubling capillary density in the border zone compared with controls, indicating efficient miRNA delivery for functional myocardial restoration [101]. In the study shown in Figure 5f, a matrix metalloproteinase (MMP)-2/9-sensitive four-arm polyethylene glycol (tetra-PEG) hydrogel system (MPGC4) was used to immobilize a carbon-dot nanocomposite electrostatically complexed with interleukin-4 (IL-4) plasmid DNA. This system not only enables in situ tracking via the fluorescent properties of carbon dots but also allows on-demand gene release after myocardial infarction, precisely correcting immune imbalance in the infarct microenvironment [88]. For non-coding nucleic acids, nanoparticle–hydrogel systems for local delivery of microRNAs (miRs) after myocardial infarction can effectively overcome the limitations of traditional transfection systems, such as high immunogenicity and poor targeting efficiency, enabling long-term sustained release and stable transfection in situ [102].

Furthermore, to achieve multidimensional synergistic repair, recent studies have focused on integrated delivery strategies. For instance, a hydrazone-linked hyaluronic acid/aldehyde laminarin self-healing hydrogel loaded with circular RNA (circRNA) nanoparticles and strontium ions has been injected intramyocardially, combined with an epicardial patch loaded with nitric oxide (NO) liposomes, achieving a gene–ion–gas tri-modal release system that overcomes the limitation of epicardial-to-endocardial diffusion for myocardial regeneration [103]. Similarly, a TA-PEG and HA-SH conductive hydrogel co-loaded with plasmid DNA nanocomplexes encoding eNOS and adipose-derived stem cells (ADSCs) enables enhanced local nitrite concentration through upregulated endothelial nitric oxide synthase (eNOS), achieving a three-dimensional coordinated repair involving gene, cell, and material-mediated electrical signaling [81].

Gene-delivery hydrogels offer the potential for sustained and localized gene expression, enabling long-term regulation of therapeutic targets during myocardial repair. However, their clinical translation remains challenged by several critical issues, including insufficient in vivo delivery efficiency, limited control over spatial and temporal gene expression, potential immunogenicity and off-target effects, long-term biosafety concerns associated with gene carriers, as well as the complexity of large-scale manufacturing and regulatory approval.

### 4.7. Stimuli-Responsive Hydrogels and Microenvironment Remodeling

Stimuli-responsive hydrogels have attracted extensive attention because they can sense pathological changes in the myocardial infarction (MI) microenvironment and achieve spatiotemporally controlled release of therapeutic factors. Among endogenous stimuli, reactive oxygen species (ROS) and acidic pH are key features of post-infarction oxidative stress, inflammation, and adverse remodeling, making them important targets for intelligent hydrogel design (Figure 5g,h). For example, ROS-responsive hydrogels based on boronic-acid-functionalized chitosan can trigger nitric oxide release under high ROS conditions, thereby restoring ROS/NO balance and alleviating ischemia–reperfusion injury [104]. In addition, ROS-responsive PVA–TSPBA hydrogels enable sustained drug release while scavenging ROS, promoting M2 macrophage polarization and angiogenesis to improve cardiac repair outcomes [105].

Beyond ROS responsiveness, acidic microenvironments in infarcted tissue have also been widely exploited to design pH-responsive systems. Jia et al. developed an alginate-based composite hydrogel with programmed spatiotemporal release, in which the pro-angiogenic small molecule UCL-TRO-1938 was rapidly released during the acute phase, while BMP-9 loaded in EGCG/Zn-coated mesoporous silica nanoparticles was released under acidic conditions during later stages, enabling sequential regulation of angiogenesis and anti-fibrotic remodeling [106]. Similarly, a methacrylated carboxymethyl chitosan hydrogel can accelerate the release of VEGF and rTFPI2 under acidic conditions, exerting anti-inflammatory, pro-angiogenic, and anti-fibrotic effects while reducing ROS-induced cellular damage [18]. Likewise, mesoporous silica nanoparticle-based miR-21-5p delivery systems can respond to acidic environments for on-demand release, promoting immunomodulation and vascular regeneration [107].

To match the dynamic progression of MI pathology, more advanced designs integrate multi-stage and spatiotemporally controlled therapeutic strategies. Injectable hydrogels capable of sequential release of pro-angiogenic and anti-fibrotic factors can target the acute injury and chronic fibrotic phases separately, enabling coordinated regulation of early vascular reconstruction and long-term remodeling improvement [106,108,109]. In addition, thermosensitive injectable hydrogels based on chitosan/dextran/β-glycerophosphate undergo rapid sol–gel transition under physiological conditions, facilitating minimally invasive delivery and cell encapsulation [110]. Recently reported thermo- and pH-dual-responsive hydrogels further enable co-delivery of tanshinone IIA and nitric oxide precursors for on-demand anti-inflammatory and pro-angiogenic therapy, highlighting the potential of multi-stimuli systems for precise myocardial regeneration [111].

Although stimuli-responsive hydrogels provide precise control over therapeutic release and microenvironment remodeling, their clinical translation is limited by several practical challenges. For example, endogenous stimuli such as ROS, pH, and enzyme levels may vary considerably among patients and disease stages, which could result in inconsistent therapeutic responses. In addition, excessive sensitivity may cause premature drug release or rapid degradation, whereas insufficient responsiveness may reduce treatment efficacy. Therefore, future studies should focus on establishing clinically relevant response thresholds and improving the stability and predictability of these systems under dynamic cardiac conditions.

## 5. Injection Strategies and Translational Applications of Injectable Hydrogels

The therapeutic efficacy of injectable hydrogels depends not only on their material properties and biological functions but also on the delivery strategy employed. Owing to the heterogeneous spatial distribution of tissue damage and the dynamic progression of pathological events following myocardial infarction, the injection route and delivery approach of hydrogels can directly influence their local retention, the distribution of therapeutic agents, and ultimately the overall therapeutic outcome [112,113].

Currently, intramyocardial injection remains the most widely studied delivery method. By directly injecting hydrogels into the infarct border zone, precise localization of therapeutic materials can be achieved, and local retention of active components in the damaged myocardium can be maximized, thereby enhancing both mechanical support and biological therapeutic effects. In animal experiments, intramyocardial injection is typically performed via open-chest surgery or catheter-assisted systems. Compared with epicardial implantation materials such as cardiac patches, injectable hydrogels can better adapt to the complex and irregular geometry of the infarcted region while avoiding additional trauma associated with extensive surgical procedures [114].

With the increasing demand for clinical translation, transendocardial catheter delivery has gradually attracted widespread attention. This strategy enables hydrogel delivery during routine cardiac catheterization procedures without the need for open-chest surgery, significantly reducing treatment trauma. Therefore, compared with biomaterials that require surgical implantation, injectable hydrogels suitable for catheter delivery are generally considered to have greater clinical application potential [115]. However, catheter delivery also imposes higher requirements on the injectability of hydrogels, including appropriate viscosity, good shear-thinning properties, and controllable gelation kinetics. If gelation occurs too quickly, the material may prematurely solidify within the catheter and cause blockage; if gelation is too slow, it may lead to material leakage, reduced local retention, and diminished therapeutic efficacy. In recent years, the clinical translation of injectable hydrogels has also made significant progress. One of the most representative cases is VentriGel hydrogel derived from decellularized porcine cardiac extracellular matrix. This material can be delivered via a catheter system and has become an injectable cardiac hydrogel that entered FDA-approved clinical trials in the United States. The results show that VentriGel has good safety and feasibility, and also demonstrates the potential to improve exercise capacity and cardiac function in patients with prior myocardial infarction [116]. In addition to VentriGel, other injectable hydrogels that have entered clinical evaluation include the alginate-based systems IK-5001 and Algisyl-LVR. Unlike these earlier clinical hydrogels, which primarily provide mechanical support by increasing ventricular wall thickness or reducing wall stress, VentriGel is a decellularized cardiac extracellular matrix (ECM)-derived hydrogel that aims to promote endogenous cardiac repair by recreating a biomimetic microenvironment. Compared with the multifunctional preclinical hydrogels discussed above, VentriGel also adopts a relatively simple acellular formulation without incorporating therapeutic drugs, cells, or genes, which may facilitate manufacturing and clinical translation [117].

Despite encouraging research progress, the large-scale clinical application of injectable hydrogels still faces many challenges. First, many hydrogel systems that perform well in small animal experiments are not necessarily compatible with existing clinical catheter platforms. Second, there is a significant discrepancy between the therapeutic time window in experimental animal models and real clinical conditions; clinical patients often receive treatment long after myocardial infarction has occurred, by which time they have already entered the stage of chronic heart failure. In addition, as components such as cells, growth factors, nucleic acids, and functional nanomaterials are continuously integrated into hydrogel systems, the product composition has become increasingly complex, placing higher demands on manufacturing processes, quality control, and regulatory approval. For such combination therapeutic products, not only must the safety of each individual component be verified, but the long-term risks of the entire delivery system must also be systematically evaluated, thereby significantly prolonging the clinical translation cycle.

At the same time, challenges associated with large-scale manufacturing, GMP compliance, sterilization, storage stability, and batch-to-batch consistency remain major barriers to clinical translation. For example, naturally derived hydrogels, such as collagen- and dECM-based systems, may exhibit variability due to differences in raw materials and processing procedures, making GMP-standardized production and quality control challenging. Moreover, sterilization and storage conditions may affect the structural integrity and bioactivity of multifunctional hydrogel systems containing proteins, drugs, or nanoparticles. Injectable hydrogels for myocardial repair must also meet clinical delivery requirements, including appropriate injectability and catheter compatibility. Therefore, successful translation requires not only therapeutic efficacy but also consideration of manufacturing feasibility and regulatory requirements [118]. In addition to biological safety and manufacturing challenges, the cost of hydrogel-based medical products should also be considered for clinical translation. The incorporation of advanced functional components, complex fabrication procedures, and strict quality control requirements may increase production costs and limit large-scale clinical application. Therefore, developing cost-effective materials and scalable manufacturing strategies will be essential to improve the accessibility of hydrogel-based therapies.

Overall, research on injectable hydrogels for myocardial repair is gradually moving from the stage of laboratory proof of concept toward clinical translation. Future efforts should focus on promoting the coordinated optimization of material design and delivery strategies, strengthening compatibility with catheter-based interventional systems, and validating long-term safety and efficacy through large animal models that are closer to real clinical conditions. With the continuous improvement of standardized production systems and precise delivery technologies, injectable hydrogels are expected to become an important component of regenerative therapy for myocardial infarction and heart failure in the future.

## 6. Future Perspectives and Challenges

Despite the significant progress achieved in injectable hydrogels for myocardial infarction (MI) repair, several challenges remain before these systems can be translated into routine clinical practice. First, many recently developed hydrogels have become increasingly multifunctional through the incorporation of nanoparticles, bioactive molecules, extracellular vesicles, or nucleic acids. Although these strategies often improve therapeutic efficacy, they also increase manufacturing complexity, quality-control requirements, regulatory burden, and production costs. Therefore, future studies should strive to achieve a better balance between therapeutic performance and clinical translatability by simplifying material design while maintaining biological functionality.

Another important challenge is the establishment of long-term biosafety. In addition to short-term biocompatibility, the biodegradation, biodistribution, immune response, and long-term toxicity of hydrogel components, particularly inorganic nanomaterials, require systematic investigation. Furthermore, most current studies are based on rodent MI models, which differ substantially from humans in cardiac anatomy, electrophysiology, and remodeling processes. Consequently, greater emphasis should be placed on validating promising hydrogel systems in large-animal models before clinical evaluation.

From a translational perspective, scalable manufacturing, standardized sterilization, batch-to-batch consistency, catheter compatibility, and cost-effectiveness are also critical considerations. Future injectable hydrogels should not only demonstrate superior therapeutic efficacy but also satisfy the practical requirements for large-scale production and regulatory approval. With continued advances in biomaterials, precision medicine, and intelligent hydrogel design, injectable hydrogels are expected to evolve toward safer, more effective, and clinically translatable therapeutic platforms, ultimately facilitating their successful application in MI repair.

## 7. Conclusions

In summary, injectable hydrogels have emerged as a promising therapeutic platform for myocardial infarction repair owing to their excellent injectability, tunable physicochemical properties, and versatile therapeutic functions. Through rational material design and functional integration, injectable hydrogels can provide mechanical support, regulate the post-infarction microenvironment, promote angiogenesis and tissue regeneration, and enable the localized delivery of bioactive agents. Although significant progress has been achieved in preclinical studies, challenges related to long-term safety, large-scale manufacturing, and clinical translation remain to be addressed. Continued advances in biomaterial engineering, intelligent design, and translational research are expected to further accelerate the clinical application of injectable hydrogels for myocardial repair.

## Figures and Tables

**Table 1:** Comparison of representative injectable hydrogel platforms for myocardial infarction repair.

Hydrogel Platform	Advantages	Limitations	Representative Functions
Natural polymers	Biocompatibility, ECM-like bioactivity	Weak mechanical strength, poor tunability	Cell interaction, inflammation regulation
Synthetic polymers	Tunable mechanics, controlled degradation	Limited biological activity	Mechanical support, stimulus response
Composite hydrogels	Multifunctionality, synergistic effects	Complex fabrication, regulatory challenges	Conductivity, drug delivery, ROS modulation

## Data Availability

No new data were created or analyzed in this study. Data sharing is not applicable to this article.
